# Effect of pomegranate (*Punica granatum*) anthelmintic treatment on milk production in dairy sheep naturally infected with gastrointestinal nematodes

**DOI:** 10.3389/fvets.2024.1347151

**Published:** 2024-02-07

**Authors:** Fabio Castagna, Roberto Bava, Ernesto Palma, Valeria Morittu, Antonella Spina, Carlotta Ceniti, Carmine Lupia, Giuseppe Cringoli, Laura Rinaldi, Antonio Bosco, Stefano Ruga, Domenico Britti, Vincenzo Musella

**Affiliations:** ^1^Department of Health Sciences, University of Catanzaro Magna Græcia, Catanzaro, Italy; ^2^Mediterranean Ethnobotanical Conservatory, Sersale (CZ), Catanzaro, Italy; ^3^Department of Health Sciences, Institute of Research for Food Safety and Health (IRC-FISH), University of Catanzaro Magna Græcia, Catanzaro, Italy; ^4^National Ethnobotanical Conservatory, Castelluccio Superiore, Potenza, Italy; ^5^Department of Veterinary Medicine and Animal Production, University of Naples Federico II, CREMOPAR, Naples, Italy; ^6^Interdepartmental Center Veterinary Service for Human and Animal Health (CISVet-SUA), University of Catanzaro Magna Græcia, Catanzaro, Italy

**Keywords:** anthelmintic resistance, gastrointestinal nematodes, sheep, ethnoveterinary pharmacology, pomegranate (*Punica granatum*), anthelmintic efficacy, milk production, animal health

## Abstract

Anthelmintic drug resistance has proliferated across Europe in sheep gastrointestinal nematodes (GINs). Sheep welfare and health are adversely impacted by these phenomena, which also have an impact on productivity. Finding alternatives for controlling GINs in sheep is thus of utmost importance. In this study, the anthelmintic effectiveness (AE) of a Calabrian ethnoveterinary aqueous macerate based on *Punica granatum* (whole fruits) was assessed in Comisana pregnant sheep. Furthermore, an examination, both qualitative and quantitative, was conducted on milk. Forty-five sheep were selected for the investigation. The sheep were divided by age, weight, physiological state (pluripara at 20 days before parturition), and eggs per gram of feces (EPG) into three homogeneous groups of 15 animals each: PG received a single oral dosage of *P. granatum* macerate at a rate of 50 mL per sheep; AG, treated with albendazole, was administered orally at 3.75 mg/kg/bw; and CG received no treatment. Timelines were as follows: D0, treatments, group assignment, fecal sampling, and AE assessment; D7, D14, D21, fecal sampling, and AE evaluation. The FLOTAC technique was used to evaluate the individual GIN fecal egg count (FEC) using a sodium chloride flotation solution (specific gravity = 1.20) and 100 × (1-[T2/C2]) as the formula for evaluating FEC reduction. Following the lambs’ weaning, milk was collected on the following days (DL) in order to quantify production: DL35, DL42, DL49, DL56, DL63, DL70, DL77, and DL84. The amount of milk produced by every animal was measured and reported in milliliters (ml) for quantitative evaluations. Using MilkoScan TM fT + foss electric, Denmark, the quality of the milk (casein, lactose, protein concentration, and fat, expressed as a percentage) was assessed. The macerate demonstrated a considerable AE (51.8%). Moreover, its use has resulted in higher milk production rates quantitatively (15.5%) and qualitatively (5.12% protein, 4.12% casein, 4.21% lactose, and 8.18% fat). The study showed that green veterinary pharmacology could be the easiest future approach to counteracting anthelmintic resistance in sheep husbandry.

## 1 Introduction

Similar to other livestock species, small ruminant farming is essential to the long-term viability of rural communities around the world (1). It also has a significant impact on national and international economics and society (2, 3). Parasitic infections are a major obstacle to the efficient production of small ruminants (4, 5), particularly in grazing sheep, which are highly vulnerable to various parasite species (6). In addition to causing poor development in young animals, lower reproductive performance, wasted organs at slaughter, and, in extreme situations, the death of the afflicted animals, parasites also result in significant losses in terms of decreased milk and meat output, both qualitatively and quantitatively (7, 8). Production losses resulting from subclinical illnesses are the primary economic consequence of parasitism (9).

The yearly economic effect of parasitic helminth infections in small ruminants in Europe is estimated to be € 443 million, with the loss of livestock output accounting for 81% of these expenditures. The yearly cost of helminth infections to dairy sheep cattle in Italy is estimated to be more than €12 million (10). Currently, parasite-related financial and agricultural losses significantly affect the farmer’s profitability (11, 12), necessitating the implementation of control programs against parasitic illnesses (13).

The use of anthelmintic drugs is the mainstay of management methods to minimize animal production losses caused by GIN infections in ruminants. Anthelmintic-resistant (AR) parasites to one or more drug classes have, however, developed as a result of the improper and sole use of these treatments (14, 15).

Many of these occurrences were noted in small ruminant GINs throughout several global locations. According to recent meta-analyses, AR is common among GINs in Europe. It has been documented in 16 European countries and 5 GIN genera: *Haemonchus*, *Teladorsagia*, *Cooperia*, *Nematodirus*, and *Trichostrongylus* (16). The findings showed that, in sheep and goats, the average farm-level prevalence of AR was 48 and 51% for benzimidazoles, 29 and 44% for macrocyclic lactones other than moxidectin, and 32 and 20% for levamisole (16, 17). There have not been many published cases of AR in small ruminants in Italy against levamisole, macrocyclic lactones, and benzimidazoles (18). Nevertheless, in 2007, there were reports of AR in small ruminants in central and southern Italy for benzimidazoles, levamisole, and ivermectin (19, 20). Further reports of AR for benzimidazoles, levamisole, ivermectin, moxidectin, and eprinomectin were subsequently made by three investigations carried out in northern Italy on sheep and goats (21, 22). Although the phenomenon has not previously happened (23), the most recent occurrence of AR was found in certain sheep farms in southern Italy for albendazole (24).

GINs are drug-resistant and pose a serious risk to sheep flocks and farmers (25). In addition, all of this may have a significant negative influence on food safety, biodiversity, and sustainability of production owing to drug residues in animal feces (26).

The growing prevalence of parasite resistance to anthelmintics, consumer worries about drug residues in food products and the environment, and the detrimental impact of preventive treatments on the development of natural immunity against helminths are just a few of the disadvantages associated with using conventional anthelmintics (27). As a result, a workable substitute must be found using predictive, preventive, and systemic medical principles that keep zootechnical and sanitary risks below a reasonable threshold (28, 29).

Since some medicinal plants possess therapeutic qualities that are also present in synthetic drugs and lack the above-mentioned adverse effects, phytotherapy could be an option. Research on veterinary phytotherapies for the management of endoparasites in sheep has been heavily pushed in recent years (30–34). Farmers and traditional healers have used phytomedicine to cure parasitism and enhance animal performance, and a large number of contemporary commercial formulations are plant-based. Despite their widespread use in ethnoveterinary medicine, only minimal scientific data support the antiparasitic properties of the majority of plant items (35).

Many small farmers and pastoralists in the Calabria region (Southern Italy) still treat sheep with GIN infections with traditional plant mixes. Pomegranate (*Punica granatum*) extract is the one that has received the most attention and has been widely used. Aqueous pomegranate (*P. granatum*) macerate has already demonstrated efficacy in *in vivo* and *in vitro* studies (31, 36, 37). Pomegranate (*P. granatum*) components with anthelmintic properties include tannins and alkaloids (31).

Although there are several studies demonstrating the anthelmintic properties of pomegranate mixtures, there are no publications testing how anthelmintic treatments with pomegranate can affect animal production. Therefore, this study evaluated how anthelmintic treatment of a pomegranate (*P. granatum*) ethnoveterinary mixture in naturally GIN-infected dairy sheep can affect qualitative and quantitative milk production.

## 2 Materials and methods

### 2.1 Study area and animals

The investigation was performed in the spring in the Mediterranean-climate Calabria region. Calabria is a region in southern Italy that has a strong agricultural and pastoral background and a sizable animal legacy (37). In this area, there are 10,113 sheep farms and 187,445 total sheep (June 2023, data provided by the BDN of the Zootechnical Registry established by the Ministry of Health at the CSN of the “G. Caporale” Institute in Teramo, Italy) (38) indicating a high prevalence of sheep breeding. After a thorough examination of these statistics, the area ranks fourth in terms of sheep breeders and fifth in terms of sheep totals throughout the country. In particular, a semi-extensive dairy sheep farm in the Province of Catanzaro (mean alt. 380 m a.s.l.) that raises its animals on mountainous pastures along the Ionian Sea coast was the research’s intended participant. The anthelmintic efficacy against GIN of a *P. granatum*-based aqueous macerate, derived from local ethnoveterinary knowledge, was evaluated *in vivo* in sheep farming. The study was conducted in the spring on 45 sheep that had not received any antiparasitic treatment for at least 6 months. The sheep used in this study were of the Comisana breed and were homogeneous in age (2 years ±0.5), body weight (42 kg ±1.8), physiological state (pluripara at 20 days before giving birth), and parasite intensity. The 45 experimental sheep were selected from 60 pluripara sheep that had had twins in the previous year. The sheep were kept on natural pasture during the day and locked in the stable at night. The prevalent species in grassland were: graminaceae (*Avena fatua, Hordeum murinum, Dactylis glomerata*, and *Dasypyrum villosum*), leguminosae (*Trifolium repens* and *Hedysarum coronarium*), and apiaceae (*Ridolfia segetum* and *Foeniculum vulgare*). The soil has a compact structure with a prevalence of clay and good water retention. Specifically, the antiparasitic effect of pomegranate-based ethnoveterinary macerate was compared with the synthetic drug albendazole. Any advantages to milk production were also evaluated in qualitative and quantitative terms. The sheep recruited for this survey were recognized by permanent markings that varied in color depending on the group they belonged to and remained the same throughout the survey. The University of Catanzaro “Magna Graecia” ethics committee, with permission number 97 of 09/10/2015, has indicated its approval of this investigation and all animal-based experimental techniques.

### 2.2 Experimental protocol for anthelmintic efficacy evaluation

The experimental protocol foresaw the collection of 60 individual fecal samples, taken directly from the fecal ampoule of the sheep, 7 days before the test (D7). The laboratory analyses were useful to verify the presence of GINs and to select the 45 suitable sheep to form study groups homogeneous in terms of parasite intensity for GINs. Three experimental groups of 15 sheep were established: *P. granatum* group (PG), albendazole group (AG), and control group (CG). PG and AG, on day 0 (D0), were treated with a single dose, administered orally, of 50 mL of pomegranate extract and 3.75 mg/kg/body weight of albendazole, respectively. CG did not receive any treatment. In all animals, individual stool samples were collected from the fecal ampoule. The dosages used in the tests are those indicated by the breeder who prepared the ethnobotanical mixture based on pomegranate and the therapeutic dosages indicated for albendazole (Sverminator® - oral suspension, Fatro) for treatments against GINs. On days 7, 14, and 21 (D7, D14, and D21), fecal samples were collected from the rectal ampulla for anthelmintic efficacy evaluation.

### 2.3 Experimental protocol for milk evaluation

In February, all sheep gave birth to the same number of lambs in the same week (±5 days). In the period between April and June, to assess the quantity and quality of milk produced, milk was taken after the lambs were weaned. Given the rearing system, to ensure the lamb’s natural development, weaning took place gradually, starting from the second week of age, and was completed by day 30. Starting on the 35th day of lactation (DL 35), the sheep were milked twice a day. Milk samples were collected from the morning and evening milking (at 05.00 a.m. and 07.00 p.m.) on days 35, 42, 49, 56, 63, 70, and 77 of lactation (DL42, DL49, DL56, DL63, DL70, and DL77). In these days, the quantities of milk produced and the milk nutrient contents were measured.

### 2.4 Preparation of pomegranate (*Punica granatum*) macerate

The anthelmintic treatment used was derived from Calabrian veterinary ethnopharmaceutical knowledge. In particular, the *P. granatum* macerate used in the study was made by an old Calabrian breeder, following centuries-old traditions that have been handed down from generation to generation. Briefly, the breeder made this ethnoveterinary remedy using 20 kg of local ripe pomegranates. In October, when *P. granatum* fruits were at peak ripeness, the fruits were collected at an altitude of 800 m above sea level in the province of Catanzaro, Calabria region, in southern Italy. Each pomegranate fruit was cut into four parts and left to macerate in 60 L of previously boiled spring water for at least 10 months. The macerate was then filtered via a cotton filter. On average, the return amounted to approximately 70% of the initial investment. For advice on dosage, the breeder who developed and used the aqueous *P. granatum* macerate throughout the years was contacted (37). A voucher was deposited at the Mediterranean Ethnobotanical Conservatory, Sersale (CZ), Italy, at access number “*Punica granatum* 114.”

### 2.5 Chemical analysis of pomegranate (*Punica granatum*) macerate

The macerate composition was investigated using liquid chromatography-electrospray ionization mass spectrometry (LC/MS-ESI). The chromatographic analysis was performed using a Dionex Ultimate 3,000 RS from Thermo Scientific (Rodano, MI, Italy). High-Resolution Mass Spectrometry (HRMS) was performed using a Thermo Scientific Q-Exactive mass spectrometer located in Rodano, MI, Italy (31).

### 2.6 Parasitological analysis

The individual fecal egg count (FEC) was determined using the FLOTAC technique with a detection limit of two eggs per gram (EPG) of feces and a sodium chloride-based flotation solution with a specific gravity of 1.200 (39). Furthermore, each group at D0, D7, D14, and D21 had a pooled fecal culture in accordance with the Ministry of Agriculture, Fisheries, and Food’s procedure (MAFF) (40). Briefly, the coprocultures were prepared with at least 50 g of feces for each group for the *in vitro* growth of the third-stage larvae (L3). For this purpose, the feces were mixed and placed in suitable containers to incubate at a temperature of 25°C for 14 days. Subsequently, the L3 was separated using the Baermann technique and identified morphologically using the morphological keys proposed by van Wyk and Mayhew (41). For GIN genera identification and percentage calculations, 100 L3 were used; if fewer than 100 L3 were found, all larvae were identified. As a result, using the total number of larvae found, it was feasible to determine the percentage of each species. The World Association for the Advancement of Veterinary Parasitology (WAAVP) guidelines for evaluating the efficacy of anthelmintics in ruminants recommend calculating the arithmetic mean of the EPG on each occasion of fecal sampling. Following these guidelines, the percent efficacy (%) of each treatment group was evaluated using the fecal egg count reduction test (FECR) at D7, D14, and D21 (42).

### 2.7 Health assessment

All animals were observed clinically by the veterinarians engaged in the study to identify potential adverse effects at an early stage. Each sheep had a thorough examination (general physical examination), paying attention to sensory status, mucous membranes, feeding, defecation, behavior, and the presence of vaginal discharge.

### 2.8 Milk analysis

After the milking of each sheep, quantitative measurements of each animal’s milk production (measured in milliliters) were made. In the laboratory, the MilkoScan TM FT+ Foss Electric, Hillerød, Denmark, was used to evaluate the milk nutrient contents: protein (%), casein (%), lactose (%), and fat (%).

### 2.9 Statistical analysis

The anthelmintic efficacy was assessed using the following formula:


$$\mathrm{FECR}=100\left(1-\left[\frac{T2}{C2}\right]\right)$$

Where T2 is the post-treatment FEC of the treated group and C2 is the mean post-treatment FEC of the untreated control group (42). This formula is based on the arithmetic mean of the control and treated groups. The protein content (%), casein (%), lactose (%), and fat (%) arithmetic means have been computed for the D35, D42, D49, D56, D63, D70, and D77 and the whole lactation period for statistical analysis. Fisher’s least significant difference (LSD) was used as a *post-hoc* analysis method in a one-way ANOVA to compare the percentages of milk produced by the animals in the three groups. STATA 10.0 software (Stata Corp., TX 77845, USA) was used for statistical analysis. GraphPad PRISM 9.5.1 (GraphPad Sofware, Inc., La Jolla, CA, USA) was used to analyze the data. The results are expressed as mean ± standard deviation (S.D). Normality was tested using the Shapiro–Wilk test. Data without normal distribution were analyzed using Kruskal–Wallis analysis of variance followed by Dunn’s tests. The Mann–Whitney test was used for the comparison of data derived from two specific groups. A repeated measures ANOVA was performed to compare the treatments at the experimental times of observation. The sphericity assumption was checked by Mauchly’s test; then, a Greenhouse–Geisser correction was performed to adjust the violation of the sphericity assumption. Tukey’s *post-hoc* test was performed for pairwise comparisons. The repeated measures ANOVA was performed with JASP (version 0.16.3). Values with a value of *p* < 0.05 were considered statistically significant.

## 3 Results

### 3.1 Pomegranate (*Punica granatum*) macerate chemical analysis

**Figure 1:**
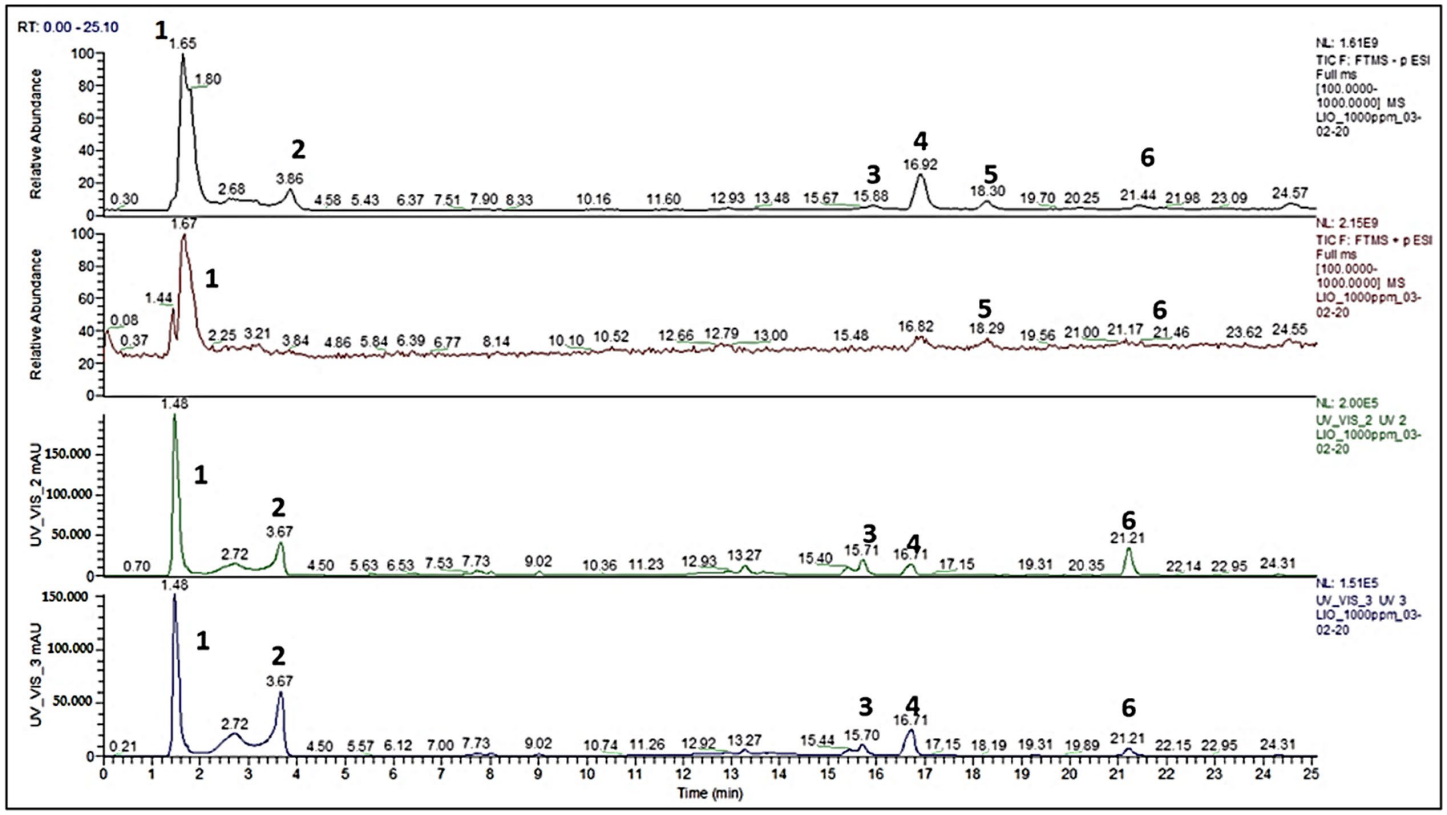
Pomegranate (*Punica granatum*) dry extract full scan analysis, by Castagna et al. (31).

To effectively fractionate the aqueous pomegranate macerate, several extraction methods and solvents were used. DCM and AcOEt, two apolar organic solvents, were used in a liquid/liquid extraction (LLE). A measure of 10% of the w/w proportion was recovered using AcOEt, while DCM LLE was mostly unsuccessful. Additionally, both EtOH and MeOH were used for the drying and filtering of the extract. Each washing produced a fraction that was qualitatively comparable to the AcOEt LLE extraction, although the recovery efficiencies were 30% w/w for EtOH and 70% w/w for MeOH. As a result, the macerate was separated based on its solubility in methanol, as was mentioned in the experimental section. The aqueous macerate and the two resulting fractions (the methanol fraction A and the insoluble residue B) were compared by LC-HRMS analysis. The aqueous pomegranate macerate was usefully fractionated using several extraction methods and solvents. Figure 1 displays the chromatographic characterization of the whole macerate. In particular, the results of the entire dry extract’s full scan liquid chromatography-high resolution mass spectrometry (LC-HRMS) analysis are shown. The results are recorded in the UV/VIS (240 nm), UV/VIS (280 nm), and ESI(+1) detection modes, respectively (31).

The greatest amount of information about the mixture’s chemical makeup is available using the ESI (−) ionization method. UV/VIS studies are complemented by mass spectra. Two separate zones may be identified in the chromatogram based on the retention times (r.t.). The molecules not firmly maintained by the stationary phase, such as simple and complex sugars (compounds 1, 2, Figure 1), make up the first area, with r.t. between 0 and min. The typical phenolic acid and ellagitannin peaks were seen in the second area, which spanned 15–25 min r.t. (compounds 3, 4, 5, and 6; Figure 1). To fully identify each typical peak seen in Figure 1, MS/MS-ESI analysis was carried out. The characterization, m/z values (obtained for LC/HRMS, ESI(−)), and structural identification of each component are shown in Figure 1. A combination of tartaric acid, glucuronic acid, mannitol, and a small proportion of an ellagitannin complex (2,3-(S)-hexahydroxyphenyl-D-glucose) make up the peak designated as (1). In Figure 1, the primary component of the methanol fraction was represented by the peak designated as (2). Peak 3, Figure 1, shows the two elagitannin derivatives valoneic acid and felligridine J; peak 4, Figure 1, shows syringic acid; peak 6, Figure 1, shows ellagic acid and ducheside A; and peak 5 shows an unknown molecule. Table 1 shows the chemical characterization of the macerated dry extract.

**Table 1:** Chemical composition of the dry extract, by Castagna et al. (31).

Peak LC–MS	m/z theoretical	m/z measured	Molecular formula	Analyte
(1)	149.0092	149.0081	C_4_H_5_O_6_	Tartaric acid
	181.0718	181.0711	C_6_H_13_0_6_	Mannitol
	193.0354	193.0347	C_9_ H_9_ O7	Glucuronic acid
	481.0697	481.0626	C_20_H_17_O_14_	2,3-(S)-hexahydroxyphenyl-D-glucose
(2)	169.0142	169.0134	C_7_H_5_O_5_	Gallic acid
(3)	288.9990	288.9992	C_13_H_5_O_8_	Phelligridin J
	469.0049	469.0050	C_21_H_9_O_13_	Valoneic acid dilattone
(4)	197.0455	197.0449	C_9_H_9_O_5_	Syringic acid
(5)	–	186.1129	C_13_H_14_O	Unknown
(6)	300.9990	300.9991	C_14_H_5_O_8_	Ellagic acid
	447.0642	447.0573	C_20_H_15_O_12_	Ducheside A

### 3.2 Parasitological studies

**Figure 2:**
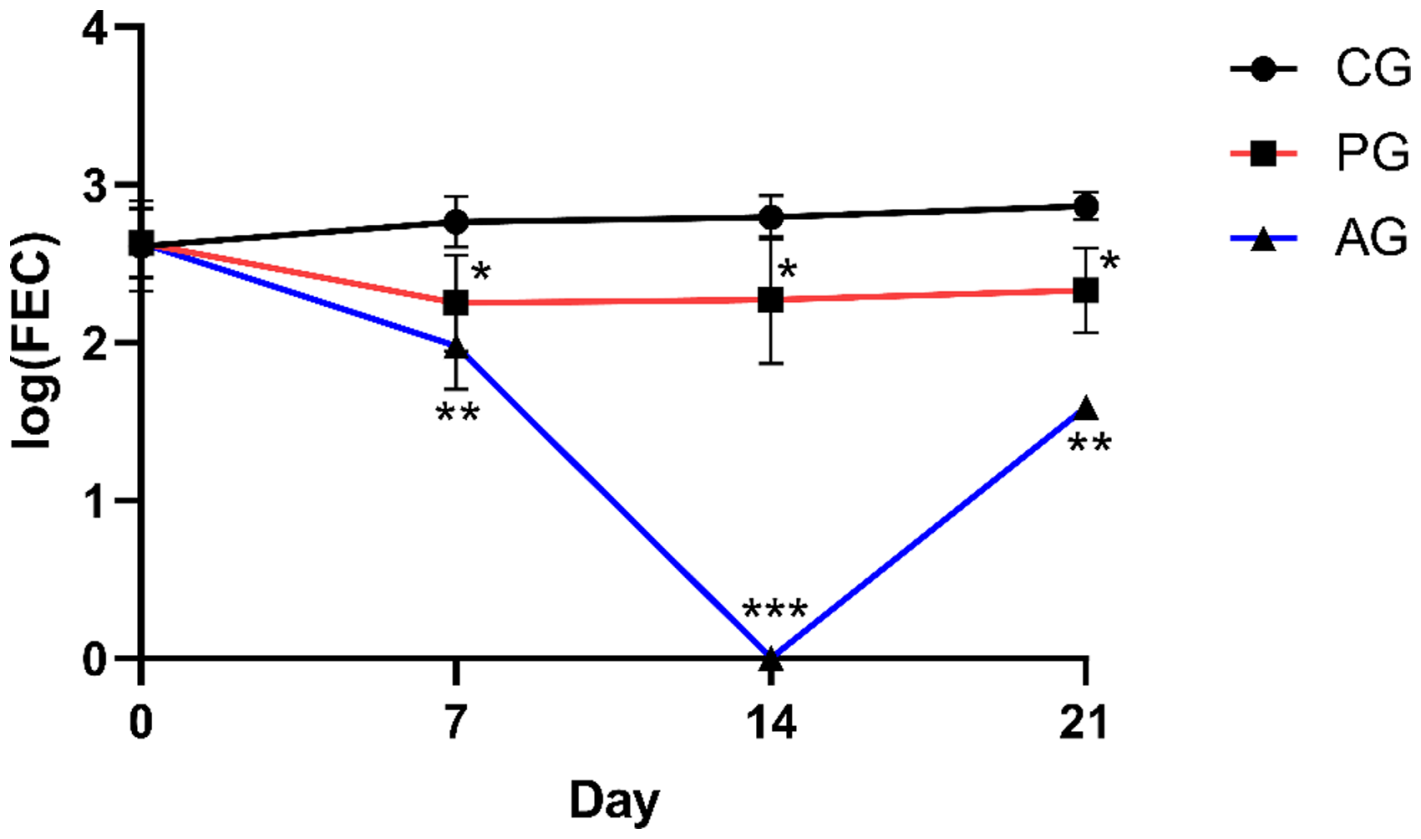
Comparison of eggs per gram. ^*^Value of *p* < 0.05 versus CG, ^**^value of *p* < 0.01 versus CG, ^***^value of *p* < 0.001 versus CG. CG, control group; PG, *P. granatum* group; AG, albendazole group.

Figures 2 3 show the results of anthelmintic efficacy obtained by comparing the macerate with albendazole and the control group.

**Figure 3:**
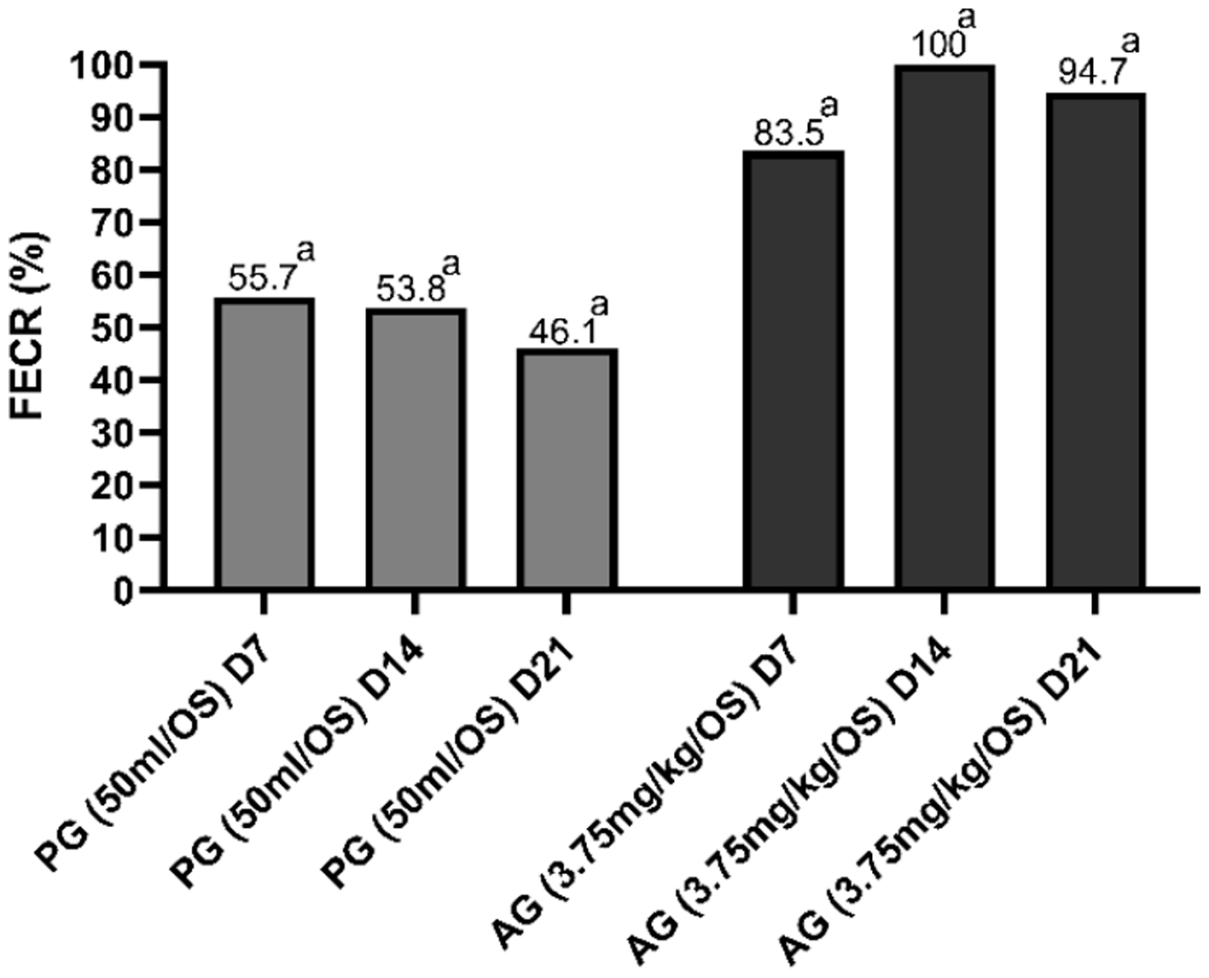
Percentage of fecal egg count reduction (FECR) in sheep. ^a^Value of *p* < 0.001 versus CG. CG, control group; PG, *P. granatum* group; AG, albendazole group.

At day 0, all three experimental groups showed the same level of FEC. After 7 days, the *P. granatum*-treated group (PG) was able to significantly reduce FEC levels compared with the control group. This reduction was also significant when the CG group was compared with the albendazole-treated group. After 14 and 21 days, both the *P. granatum*-treated group and the AG group showed a significant reduction of EPG compared with the control group (37).

In the PG group and AG group, the percentage of FECR was statistically significant (*p* < 0.001) compared with the control group on days 7, 14, and 21. The genera of GINs detected before treatments (D0), expressed as a percentage (%), were as follows: *Teladorsagia* 42, 45, and 35%; *Trichostrongylus* 32, 45, and 35%; *Haemonchus* 18, 13, and 21%; *Chabertia* 8, 6, and 3% for CG, PG, and AG, respectively (37). GINs genera detected in all groups before treatments, expressed as a percentage (%), were *Teladorsagia* 45, 32, and 44% (D7); 47, 43, and 0% (D14); 44, 48, and 0% (D21), for CG, PG, and AG, respectively. *Trichostrongylus* 30, 38, 40% (D7); 28, 32, and 0% (D14); 35, 35, and 0% (D21), for CG, PG, and AG, respectively. *Haemonchus* 11, 18, and 15% (D7); 14, 11, and 0% (D14); 8, 9, and 0% (D21), for CG, PG, and AG, respectively. *Chabertia* 14, 12, and 1% (D7); 11, 14, and 0% (D14); 13, 8, and 3% (D21); for CG, PG, and AG, respectively (37). The coproculture results in PG-treated patients revealed no appreciable changes in the ratio of gender percentages detected before treatment and after treatment.

### 3.3 Health assessment

Despite the presence of *Haemonchus contortus* among the identified GIN genera, anemia was not evident, presumably because the percentages were low. All animals completed the study. No side effects were observed during clinical observation of the animals tested after treatment with aqueous pomegranate macerate and albendazole. All sheep enrolled in the study completed their pregnancies; no premature births were observed, and the lambs born had no health problems.

### 3.4 Milk analysis

Tables 2 3 report the associated qualitative and quantitative evaluations of milk, with the mean output expressed in milliliters (ml) relative to the various days of lactation (DL) of the treated and untreated groups.

**Table 2:** Quantitative milk production, results related to the comparison between the anthelmintic efficacy of the pomegranate (*Punica granatum*) aqueous extract with albendazole and milk production.

Groups	Milk production (ml)											
	DL35	DL42	DL49	DL56	DL63	DL70	DL77	DL84	Mean	SD	*p*-value	Increase (%)
CG	1,180	1,160	1,150	1,080	970	880	670	460	943.7	247.4		
PG	1,360	1,340	1,320	1,300	1,100	1,000	760	560	1,092^*^	284.3	<0.05	15.5
AG	1,480	1,380	1,380	1,340	1,200	1,100	860	660	1,175^**^	272.4	<0.01	25.1

Asterisks indicate statistical significance versus CG. PG-treated *P. granatum* group; AG-treated albendazole group; CG-untreated group.

**Table 3:** Qualitative milk production, results related to the comparison between the anthelmintic efficacy of the pomegranate (*Punica granatum*) aqueous extract with albendazole and milk production.

Milk components (%)	Groups	Day lactating (DL)										
		DL35	DL42	DL49	DL56	DL63	DL70	DL77	DL84	Mean	SD	*p*-value
Protein	CG	4.4	4.7	4.8	4.7	4.7	4.8	4.9	5.3	4.78	0.2419	
	PG	5.4	4.9	5	5	4.7	4.9	5.6	5.5	5.12^*^	0.3100	<0.05
	AG	4.9	5.1	5.1	5.1	4.7	5.3	5.2	5.7	5.13^*^	0.3284	<0.05
Casein	CG	3.4	3.6	3.9	3.8	3.8	4	4.2	4.6	3.91	0.1951	
	PG	3.6	4.1	4.1	4.1	3.8	4.3	4.2	4.8	4.12^*^	0.2530	<0.05
	AG	4.3	3.9	4.1	4	3.9	4.1	4.7	4.7	4.21^*^	0.2957	<0.05
Lactose	CG	4.9	3.6	4.4	4.5	4.1	4.1	3.2	3.6	4.05	0.4452	
	PG	4.8	3.4	4.5	4.3	4.4	4.2	4.3	3.8	4.21^*^	0.3548	<0.05
	AC	4.7	4.4	4.3	4.3	4.3	4.2	4.4	4.4	4.37^*^	0.2663	<0.05
Fat	CG	5.4	6.8	7.6	7.3	8.5	8.1	8.3	8.3	7.53	0.3458	
	PG	6.5	8	8.2	8.3	8.1	8.6	8.3	9.5	8.18^*^	0.2736	<0.05
	AG	7.7	8.5	9.5	8.5	8.5	8.6	9.2	9.9	8.8^**^	0.3879	<0.01

Asterisks indicate statistical significance versus CG. PG treated pomegranate aqueous extract group; AG treated albendazole group; CG % untreated group.

**Figure 4:**
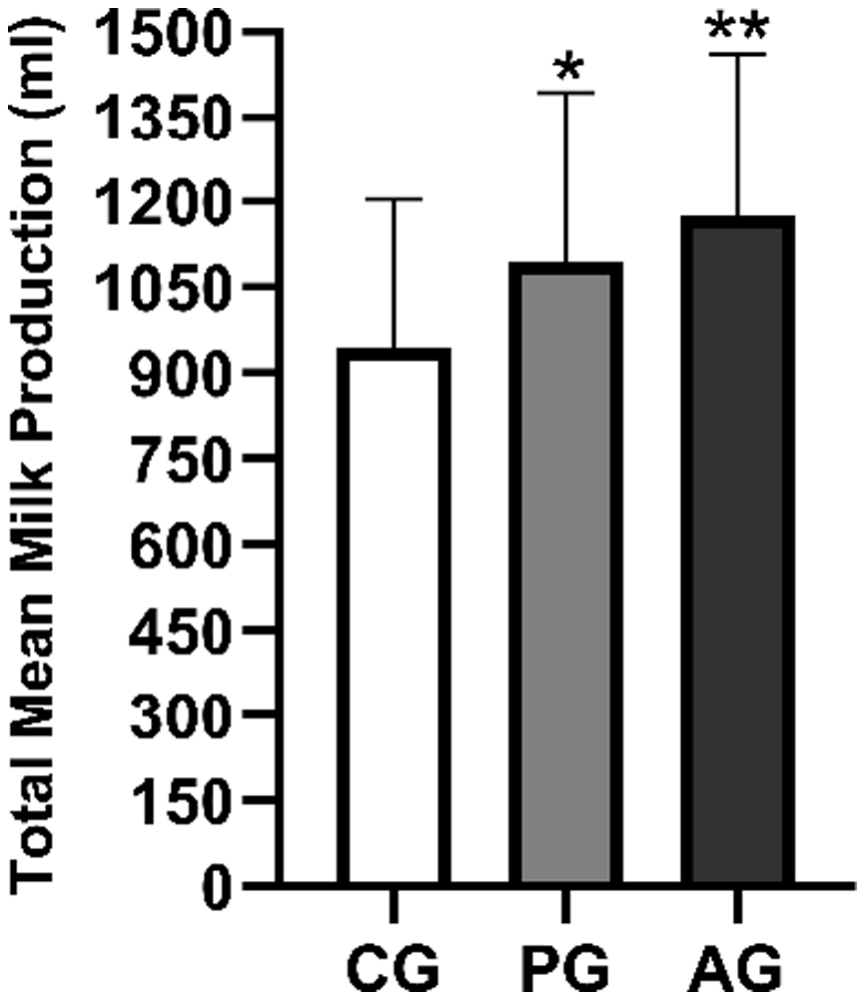
Mean of quantitative milk production in the three study groups for the duration of the entire study. ^*^Value of *p* < 0.05 versus CG, ^**^value of *p* < 0.01 versus CG. CG, control group; PG, *P. granatum* group; AG, albendazole group.

Figure 4 represents the quantitative yields in the form of histograms.

The mean milk yield in the PG group was significantly higher (value of *p* < 0.05) than that of the control group. The albendazole-treated group also showed higher average milk production than the control group but was not statistically significant (value of *p* > 0.05) compared to PG.

**Figure 5:**
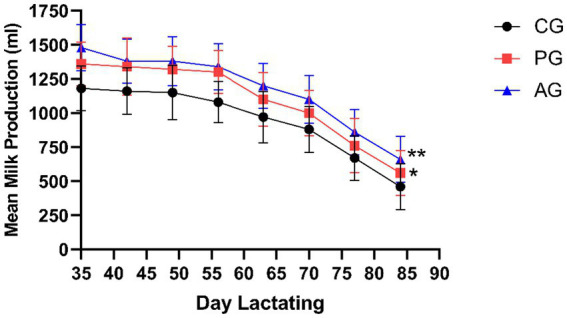
Mean milk production (ml) on lactation days (DL) 35, 42, 49, 56, 63, 70, and 77. ^*^Value of *p* < 0.05 versus CG, ^**^value of *p* < 0.01 versus CG. CG, control group; PG, *P. granatum* group; AG, albendazole group.

Figure 5 shows the average milk production (ml) on lactation days (DL) 35, 42, 49, 56, 63, 70, and 77 in the form of performance curves.

**Figure 6:**
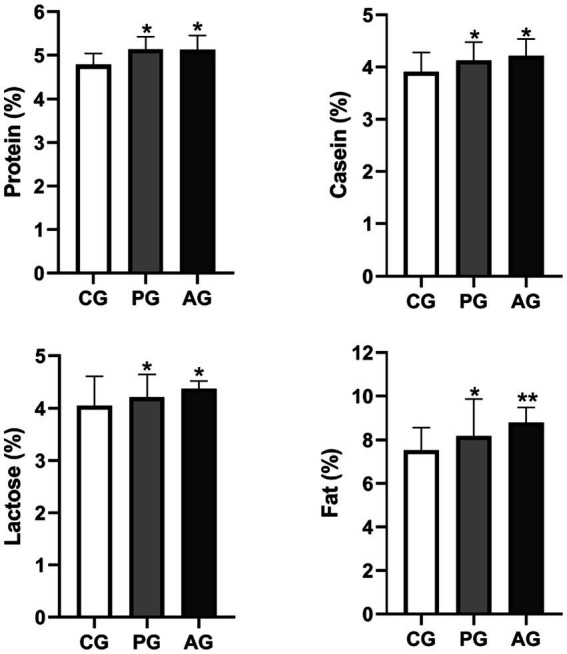
Qualitative milk composition (%). ^*^Value of *p* < 0.05 versus CG, ^**^value of *p* < 0.01 versus CG. CG, control group; PG, *P. granatum* group; AG, albendazole group.

Figure 6 represents the qualitative evaluation of milk relative to the percentage of protein, casein, lactose, and fat in the study groups.

The percentage of protein, casein, lactose, and fat was significantly higher in the PG-treated group than in the control group. The albendazole-treated group, in turn, had a higher percentage of all components of the milk under test than the control group. On the other hand, the difference in the percentage of protein, casein, and lactose was not statistically significant when compared between the PG group and the AG group.

## 4 Discussion

Several studies highlight the anthelmintic efficacy of pharmacological preparations based on pomegranate parts, e.g., leaves (43, 44) and peels (43, 45) in small ruminants. In particular, Gadhave et al. (46) demonstrated an efficacy (FECR%) of 69.4% on day 15 and 73.7% on day 28 with an ethanolic extract based on pomegranate peels (200 mg/kg b.wt for the first 5 days and on the 16th day) in goats naturally infected with GINs. However, there are no studies evaluating the anthelmintic efficacy of ethnoveterinary preparations based on the whole pomegranate fruit that also assess the influence of these treatments on milk yields. Therefore, through this study, it was possible to confirm the anthelmintic efficacy of an ethnoveterinary mixture based on the whole pomegranate fruit in sheep naturally infected with GIN and how these treatments could affect milk production and milk components. In particular, a single administration of pomegranate macerate showed a mean efficacy (FECR%) of 52.5% (D7 55.7, D14 53.8, and D21 46.1). These results were inferior to treatment with albendazole, which gave a mean efficacy (FECR%) of 92.7 (D7 83.5, D14 100, and D21 94.7). Compared to the control group, there was a significant reduction in FEC. The reduction in FEC induced by pomegranate was less than that determined by albendazole compared to the control group, where the difference was significant to a greater extent. These results are in line with those conducted in previous studies in which the same preparation was used. The reduction in FEC induced by pomegranate was less than that determined by albendazole when compared to the control group, where the difference was statistically significant to a greater extent (*p* < 0.001). The results obtained are in line with those conducted in previous studies in which the same preparation was used. In particular, in the study by Castagna et al. (36) in which the *in vivo* efficacy of this ethnoveterinary preparation was evaluated, an average efficacy (FECR%) of 44.9% (D7 50.2, D14 44.3, and D21 40.4) was observed, compared to other ethnoveterinary preparations based on *Artemisia campestris* (average FECR 11.5%) and *Salix caprae* (average FECR 1.8%). Castagna et al., (37), observed a mean efficacy (FECR%) of 52% (D0 56.2, D14 53.2, and D21 45.7) compared to the group treated with ivermectin at conventional dosages (mean FECR 90%). Importantly, in the first studies conducted by Castagna et al. (32), the same mixture had an *in vitro* efficacy of 89.3 and 99.3% at a concentration of 0.005 mg/mL and 1 mg/mL, respectively. Similar results were observed in the *in vitro* studies of Aliyi et al. (47), which observed efficacy of a methanolic pomegranate preparation of 49.33 and 94.63% against sheep *H. contortus* at concentrations of 0.1 mg/mL and 1 mg/mL, respectively. The results of this *in vivo* study highlight the differences between *in vitro* and *in vivo* test results and underline the importance of *in vivo* anthelmintic efficacy tests for the evaluation of natural preparations that can be used to treat intestinal parasites. The special feature of this study was the examination of milk both quantitatively and qualitatively after anthelmintic treatment with the pomegranate mixture. In the pomegranate-treated group, an increase in milk yields (15.5%) was recorded compared to the control group. In the evaluation of milk components, an increase in protein of 0.34%, casein of 0.21%, lactose of 0.16%, and fat of 0.65% was observed in the pomegranate-treated group compared to the control group. An increase in yield of 25.1%, protein of 0.35%, casein of 0.3%, lactose of 0.32%, and fat of 1.27% was found in the albendazole-treated group compared to the control group. Unfortunately, it is not possible to compare the study data with other similar research, as there are no studies in the literature that have evaluated the benefits of the anthelmintic activity of pomegranate on milk. Argov-Argaman et al. (48), in a study that dietary pomegranate peel had significant effects on the milk production of lactating sheep. In particular, sheep-fed pomegranate peel had higher milk production, fat, protein, and lactose content than controls. However, Valenti et al. (49) observed that the inclusion of pomegranate pulp in the diet of grazing ewes did not affect milk production or composition. This highlights that the increase in milk production and composition results from the anthelmintic efficacy of the aqueous pomegranate macerate, also demonstrated in previous studies (31, 36, 37), which also positively influences milk production, both in terms of quantity and quality. This is in line with what has been shown in studies conducted by some of the authors of this study with the administration of synthetic anthelmintics (50–54). Anthelmintic treatments against GINs positively influence milk production in dairy sheep (50–54), and treatments in the peripartum period should be used in GIN control strategies in sheep farming (55, 56). Therefore, this ethnoveterinary preparation, although a natural product derived from ethnoveterinary knowledge, represents a promising remedy with proven efficacy. Its anthelmintic efficacy is certainly not at the level of the synthetic product, but its use should nevertheless be taken into account in anthelmintic treatments and control strategies involving a virtuous rotation of veterinary drugs. According to Bosco et al. (34), the identification of potentially useful ethnoveterinary plant species is crucial for sustainable and current pasture management. Through proper health management of sheep farming, based on accurate parasitological diagnosis, good pasture management (subdivision of plots, pasture rotation with other species, etc.), and the use of bioactive fodder, the administration of synthetic anthelmintic drugs could be reduced to an acceptable threshold from a One-Health perspective. In this context, green veterinary pharmacology could represent the future approach to combating AR in small ruminants (48), while promoting animal welfare and health. These objectives would also be pursued by obtaining the consensus of the final consumer, who is increasingly attentive to the environment and to farming systems that favor natural and non-synthetic pharmacological treatments. The public’s desire for a decrease in the use of synthetic chemicals in agriculture and the general promotion of organic farming systems, particularly in the European Union and partner countries, align with the potential application of natural bioactive compounds to control GIN infections in grazing ruminants.

## 5 Conclusion

The *P. granatum* macerate produced very compelling effectiveness findings for a natural mixture. The parasite control offered could make it possible to think about incorporating it into programs for therapeutic management. In reality, it may assist in enhancing animal welfare and health as well as slowing down the phenomenon of anthelmintic resistance and its environmental effects. In conclusion, this research contributes to the growing field of green veterinary pharmacology, a field of veterinary medicine that must be adopted to make animal husbandry sustainable in a world where environmental pollution—which is also a result of careless drug use—is a constant threat.

## Data availability statement

The raw data supporting the conclusions of this article will be made available by the authors, without undue reservation.

## Ethics statement

The animal studies were approved by Ethical committee of the University of Catanzaro “Magna Græcia” with approval number 97 of 09/10/2015. The studies were conducted in accordance with the local legislation and institutional requirements. Written informed consent was obtained from the owners for the participation of their animals in this study.

## Author contributions

FC: Conceptualization, Data curation, Formal analysis, Investigation, Methodology, Writing – original draft, Writing – review & editing. RB: Conceptualization, Investigation, Writing – original draft, Writing – review & editing, Data curation, Formal analysis, Methodology. EP: Conceptualization, Supervision, Validation, Writing – original draft, Writing – review & editing, Methodology. VaM: Formal analysis, Investigation, Writing – review & editing, Methodology. AS: Formal analysis, Investigation, Writing – review & editing. CC: Formal analysis, Investigation, Writing – review & editing. CL: Formal analysis, Writing – review & editing, Investigation. GC: Methodology, Supervision, Validation, Conceptualization, Writing – review & editing. LR: Methodology, Supervision, Validation, Conceptualization, Writing – review & editing. AB: Conceptualization, Data curation, Formal analysis, Investigation, Methodology, Writing – review & editing. SR: Data curation, Formal analysis, Writing – review & editing, Software. DB: Conceptualization, Supervision, Validation, Methodology, Writing – review & editing. ViM: Conceptualization, Supervision, Validation, Methodology, Writing – review & editing.
